# Hope and connection: the experience of family caregivers of persons with dementia living in a long term care facility

**DOI:** 10.1186/1471-2318-13-112

**Published:** 2013-10-20

**Authors:** Wendy Duggleby, Dawn Schroeder, Cheryl Nekolaichuk

**Affiliations:** 1 Faculty of Nursing, University of Alberta, Alberta, Canada; 2 Division of Palliative Care Medicine, Department of Oncology, University of Alberta, Alberta, Canada

**Keywords:** Hope, Connection, Caregivers, Dementia

## Abstract

**Background:**

Hope is a psychosocial resource that is essential for the psychological, spiritual, and physical well-being of family members caring for persons with dementia. A significant positive relationship has been found between hope and well-being in family caregivers of persons with dementia living in the community. However, the hope experience of family caregivers of persons living with dementia in long-term care (LTC) facilities has not been explored. The purpose of this study was to explore the hope experience of family caregivers of persons with dementia living in a LTC facility.

**Methods:**

Twenty-three open-ended face to face interviews were conducted with 13 family caregivers of residents with dementia in a LTC facility. Family was broadly defined to include relatives and friends. Seven of these participants also reflected on their hope in diaries over a two week period. Interview transcripts and journal texts were analyzed using Thorne’s interpretive description approach.

**Results:**

The over-arching theme was “hope and connection”. Participants lost hope and felt despair when they perceived they were unable to connect with their family member in the LTC facility. They regained their hope when a connection could be made. Several sub-themes were identified including: accepting where we are, living life in the moment, believing in something, standing together, and balancing dual worlds.

**Conclusions:**

Hope was important and essential for family caregivers of persons with dementia residing in a LTC facility. The overarching theme of “hope and connection” underscores the importance of maintaining relationships and connection between family members and the person in LTC. Given the paucity of hope research conducted within this population, the study findings provide a foundation for future research.

## Background

Poor physical and mental health outcomes in families of persons in LTC settings are similar to those associated with family caregiving of persons in the home [1]. Moreover, family caregivers of persons with dementia have higher levels of depression than caregivers of persons who are cognitively intact [2]. Thus, family caregivers of persons with dementia face poor long-term health outcomes if they do not receive adequate support while caregiving.

Hope is a largely neglected psychosocial resource that is essential for the psychological, spiritual, and physical health of family caregivers of persons with dementia [3]. It is a dynamic experience of possibilities for a better future that helps family caregivers deal with the caregiving experience [4]. Only two published studies have focused on hope in family caregivers of persons with dementia [3,5]. The first, a study of 88 caregivers of persons with dementia, reported a positive significant relationship between hope and well-being of the family caregivers [5]. In the second study, a grounded theory study of hope in 17 family caregivers of persons with dementia living in the community [3], participants described psychologically leaning on their hope every day to deal with their caregiving issues and to continue caregiving. They connected with others and their faith to help them to see positives in their lives and possibilities.

Although these two studies provide some understanding of the hope experience of family caregivers of persons with dementia, the LTC setting creates unique challenges for family caregivers of persons with dementia. For example they experience power inequities with LTC staff and also need to advocate for their family member [6]. These unique challenges suggest that there may be a difference in the hope experience of informal caregivers of persons with dementia residing in LTC versus those living in the community. Family caregivers provide both direct physical and indirect care [1,7-9], and play an important role in providing care to dementia patients in LTC [10,11]. Understanding hope is vital to providing support to this population. Thus the purpose of this study was to explore the hope experience of family (broadly defined as family or friend) caregivers of persons with dementia residing in a LTC facility.

## Methods

### Design

In this qualitative study, Thorne’s [12] interpretive description methodological approach was used to achieve a more in-depth understanding of the concept of hope in family caregivers of persons with dementia living in LTC. Interpretive description is a qualitative research approach that provides a thematic or integrative description of a phenomenon. Using this approach, elements and patterns of a common issue are explored to render an understanding of them, at the same time honoring their inherent complexity [12]. The term approach is used rather than design as interpretive description does not have a prescriptive set of steps. Rather, it is an approach that guides all aspects of the research from the generation of the question to the analysis. Essential elements of interpretive description as described by Thorne [12] are: a) epistemological integrity from research question to findings, b) representative credibility such as using more than one data source, c) analytic logic which includes rich and thick descriptions from the data and using an audit trail, d) interpretive authority in that the findings are substantiated with data examples, e) moral defensibility in that there is a sound reason for conducting the research, f) pragmatic obligation in that the findings can be used in practice, g) contextual awareness so that the findings are interpreted within a context and h) probable truth for the study participants within a context. Interpretive description is best suited for research questions arising from complex, clinical concerns that seek to yield practical applications [12]. It is a useful approach as it values and attends to multiple realities that are contextually bound. Thus interpretive description as a methodological approach can provide insight into the family caregivers’ hope experience when caring for a family member or friend with dementia in LTC. This study was approved by the University of Alberta’s Health Research Ethics Review Board, in Edmonton, Alberta.

### Sample

Thorne [12] suggests that both purposive and theoretical sampling be used. Purposive sampling was used to recruit informal caregivers identified as family of residents with dementia in a LTC facility in Edmonton, Alberta. A social worker at the facility initially contacted persons who met the following inclusion criteria: 1) family or friend caregiver of a person with dementia living in LTC, 2) male or female, 3) 18 years of age or over, and 4) English speaking. A research assistant subsequently contacted those individuals who verbally consented to being approached with more information about the study. Purposive sampling was utilized to ensure there was diversity in the sample in regard to age, gender, and length of time the resident had resided in a LTC facility. Theoretical sampling occurred once data collection was underway to achieve an in-depth understanding of the emerging findings. Theoretical sampling is the process of identifying additional sampling criterion to inform possible variation within concepts that have emerged from the data analysis [12]. For example as data analysis progressed, participants with different relationships (sister, daughter, son etc.) with the persons in the LTC facility were sampled to inform the emerging theme of “hope and connection”.

### Data collection

Thirteen out of 20 eligible participants consented to take part in the study. These participants were associated with 10 of the LTC facility’s residents. Reasons given by the seven who declined to participate included: disinterest, scheduling difficulties, family crises, poor health, and other family caregiver commitments.

A trained research assistant obtained written informed consent from the 13 participants and a copy of the signed consent form was given to each participant. Data collection for this study involved qualitative interviews and diaries. Following the consent process, participants completed a demographic form. Face-to-face audio-taped interviews were held either in the person’s home or in a meeting room/quiet corner of the atrium in the LTC facility. Participants were also given a journal and asked to write their thoughts on their experiences of hope over a two-week period following the first interview. The interviews lasted approximately 60–90 minutes using an open-ended interview guide, with the following questions: a) tell me about hope, b) what gives you hope, c) what kind of things change (increase or decrease) your hope, and d) what can others do to help you have hope.

The research assistant contacted the participants approximately two weeks after the first interview to arrange a time to pick up the journal and to schedule a second interview. Seven of the 13 participants wrote in their journal and participated in second interviews. Second interviews were conducted to explore in-depth what was discussed in the first interview. Other reflections on the experience of hope that may have been triggered from the first interview were also elicited. Some participants who declined a second interview stated that they felt they did not have anything more to say about their hope experience and others did not return the research assistant’s telephone message. Three participants were interviewed a third time after the initial data were analyzed to further understand the overall preliminary findings of the study. As the findings reflected an interpretive process, they were not changed, but additional in-depth data were collected through the third interview on the participants’ hope experience. In total 23 interviews were completed.

### Analysis

Data analysis occurred concurrently with data collection using Thorne’s [12] interpretive description approach. All interview recordings and journals were transcribed verbatim by an experienced transcriptionist and then checked for accuracy. Interview and journal data were analyzed together. Initial codes were developed by collecting data bits with similar properties. These initial codes were then developed into concepts, themes, and potential subthemes. Constant comparative analysis was also used to ensure that data were examined thoroughly for similarities and differences among all participants. From the categories, themes that best illustrated the participants’ experiences of hope, including what fostered or hindered their hope were identified. Demographic data were entered into SPSS, v 19 and analyzed using descriptive statistics.

Scientific rigor for the qualitative data was achieved through procedures to ensure trustworthiness of data, such as checking transcripts for accuracy, using participants’ words as much as possible and keeping an audit trail.

## Results

All 13 participants were Caucasian and the largest group (46%) were daughters. The overall sample included six daughters, two sons, three spouses (one husband and two wives), and two friends. Three daughters were the daughters of one resident and the two friends of a resident were married to each other. More than two thirds (69%) of the participants were female. The majority of participants (69%) were married or living in a common-law relationship, while 15% were divorced, 8% were single, and 8% were widowed. Most participants were retired, educated beyond high school, and lived in an urban setting. The median income was between $40,000 and $49,999 per year. Several informal caregivers were coping with chronic medical conditions of their own while caring for those in long-term care. Table 1 presents additional demographic data.

**Table 1:** Demographic characteristics of participants

**Variable**	**Mean (SD), range**
**Caregiver age in years**	67.1 (7.8), 53 to 82
**Education in years**	14.9 (2.8), 12 to 20
**Perception of health in relation to others the same age, where 1 is poor and 5 is excellent**	4.04 (0.8), 1 to 5
**Length of time caregiving in years**	4.6 (3.9), 1 to 15
**Age of family member/friend in LTC (in years)**	87.2 (8.5), 73 to 97
	**Frequency**
**Gender of caregiver**	
Female	9 (69.2%)
Male	4 (30.8%)
**Caregiver’s place of residence**	
Urban	10 (76.9%)
Rural	3 (23.1%)
**Caregiver’s ethnic origin**	
Caucasian	13 (100%)
**Caregiver’s marital status**	
Single	1 (7.7%)
Married	5 (61.5%)
Divorced	2 (15.4%)
Widowed	1 (7.7%)
Common Law	1 (7.7)
Missing	3 (23%)
**Caregiver’s employment status**	
Retired	9 (69.2%)
Employed	1 (7.7%)
Unemployed	0
Other	2 (15.4%)
Missing	1 (7.7%)
**Relationship to family member/friend in Long-Term Care (LTC)**	
Wife	2 (15.4)
Husband	1 (7.7%)
Daughter	6 (46.1%)
Son	2 (15.4%)
Friend	2 (15.4%)
**Health of the caregiver**	
No medical conditions	4 (30.8%)
One medical condition	4 (30.8%)
Three or more medical conditions	5 (38.4%)
**Do others help with caregiving? Yes or No**	
Yes	7 (54%)
No	3 (23%)
Missing	3 (23%)
**Frequency of caregiver visits**	
Up to once a week	3 (23.1%)
1.5 to 3.5 times per week	3 (23.1%)
4 times or more per week	7 (53.8%)
**Gender of LTC resident ( n=10)**	
Female	7 (70%)
Male	3 (30%)
**Dementia diagnosis of resident in LTC**	
Alzheimer’s disese	1 (10% )*
Other dementias	10 (100%)*
*One resident was diagnosed with mixed dementia as well as Alzheimer’s	
**Stage of dementia**	
Early to moderate	4 (40%)
Moderate to advanced	6 (60%)
**Number of years since dementia diagnosis**	
1 to 2 years	4 (40%)
3 to 5 years	3 (30%)
6 to 8 years	2 (20%)
10 years or more	1 (10%)
**Years resident in current LTC facility**	
Less than 1 year	3 (30%)
1 to 2 years	4 (40%)
2 to 4 years	2 (20%)
5 years of more	1 (10%)

### Context

The findings of the participants’ hope experiences were interpreted within the physical and psychosocial context of their caregiving experiences. Participants provided a glimpse into a dual world where their sense of self and time changed upon entering the LTC facility to visit a resident. They described their hesitation at the door of the LTC facility, as they were unsure what they would find due to the changing perceptions of reality of those they visited. For instance, one individual commented on how her father was sometimes in his “*own little world…been on a trip here, or a trip there, and…been busy taking meetings somewhere*”. Another individual spoke of how his mother at times recognized him during a visit and “*all of a sudden she might say to me, do you think my son’s coming down today? Who does she think is there – my father? He’s been passed away about 20 years ago”.* Several participants described the deterioration of the residents’ cognitive abilities with one daughter commenting on it being “*a living death*” for the resident.

Several informal caregivers who spoke of their commitment to visit a family member on a regular basis voiced appreciation for LTC facilities that provide specialized care in a safe environment. As one daughter said: *“I feel fortunate that we have this for her, and, you know, that at least we have these kinds of facilities when they get to this, because it would be very dangerous to keep her in my home”.* There was also a sense of duty among all of the caregivers to be an integral partner in the care of their family member. The responsibility of visiting certain times per week and handling the family member’s affairs outside of care was referred to by one son as “*a, uh, permanent part-time job! You know, to a certain degree”.* For some, there was an apprehension about travelling too far away from the LTC facility in case something happened to their family member’s health. One son spoke of how his mother’s health affected his decision to go skiing with others outside of the city as he said: “*I don’t know if I can go on these tours bec-because they’re by bus, and, uh, if something happened here, I have to be able to get back”*.

This context reflected the commitment of the participants’ to continue caregiving and maintaining a connection with their family member residing in a LTC facility. It also reflected the ongoing stress and feelings of uncertainty associated with their family members’ disease progression. It is within this context that they described their hope experience.

### Descriptions of hope

Within the context of their caregiving experience, participants shared their descriptions of hope. Hope was described as the possibility of a *“better tomorrow*” that was different from the participants’ current reality and an essential part of life. As one participant said: “…*what hope is,… it’s really a desire for something to be different from the reality of what is”.* The participants described positive outcomes associated with hope. For example one participant said: “*hope is what keeps people living, keeps people moving, keeps people pushing and striving for, uh, better, or as good as*”, while another stated: “*hope is life, I guess. Cause without it, you have no quality of life*”.

Hope was also described in relation to the dual worlds of the participants. They described their own hope as hope for a healthy and peaceful existence. As one participant stated: “In my life? Hope for a long, peaceful and healthy life….and-and not ending up in the same way….” At the same time their descriptions of hope and hopelessness were often associated with the loss of their ability to connect in some way with the person with dementia. As one participant described the deterioration of their father’s ability to connect with them: “You lose them. Yeah that’s right; its-it’s a tremendous loss.” Their hope and hopelessness were associated, as one participant suggested, with waves of feelings of loss: *“… and one must continually come and deal with waves of loss.* Despite these substantive losses, participants were committed to caring for and connecting with the person with dementia. This emerged from the data as an overarching theme of “hope and connection”.

#### Hope and connection

The over-arching theme from the data collected was hope and connection with five inter-related sub-themes: accepting where we are, living life in the moment, believing in something, standing together and balancing dual worlds. Although presented separately these sub-themes are overlapping and interconnected.

The focus of the participants’ hope for themselves was that they could continue to be present and experience a connection with their family member or friend residing in the LTC facility. Although several participants expressed a lack of hope for improvement in the course of dementia, they specifically expressed hope for an ongoing connection with their family member. As one spouse said: “*every day…she’ll be alert and responsive…and I guess hope is that she will stay like that for a long time”.* Hope for a connection was greatly influenced by the illness progression; with respect to her father’s illness one daughter said: “*I sure hope he’ll know who I am right to the end”.*

Connections between participants and family members were not always referred to as a verbal recognition. For example, one participant whose friend could not recognize him or communicate verbally any longer, spoke of ways he learned to connect through touch and other non-verbal signs: “*because [J] doesn’t communicate verbally, but she does communicate physically with her eyes and things, you know if you’ve upset her…you know, just to touch her on the back or the side, on her arm or her shoulder, to say you’re there, and she responds to that – doesn’t she – when we go in, she – she responds”.*

The participants’ hoped that in connecting with their family member it would improve the quality of life or well-being of the resident. As one participant said: “*So you hope that you can somehow give them some happiness”.* Happiness for persons living with dementia in the LTC facility was also expressed as moments of joy and comfort. This was eloquently expressed by one daughter who said: “*your hope is that you make it as comfortable for them as you can in your power, that you can find the best care that you can, and, um, not that you hope that she’s going to have memories of nice visits; that’s not going to happen. And I always knew that, but just maybe for the moment that she enjoys her time”.* The participants also hoped that by being present and caring for their family member, their family member would have a peaceful death. As one daughter said: “*When I think of the word HOPE in relation to Dad, I know he didn’t want to end up with his memory failing and being dependent on others for his care. So my hope is he will pass peacefully in his sleep without losing total dignity”.* The participants experienced hope in the relational space they shared with the resident in the LTC facility. When no connections were made, they lost hope and felt despair; when connections were made, they regained their hope.

### Accepting where we are

Participants described needing to accept their own situation as well as the residents’ situation in order to move forward and connect to that person in a different way. For example, accepting that the cognitive abilities of their family member or friend would not improve helped some participants reframe their hope. As one participant said: *“…you know, when you know that there isn’t hope that he’s going to suddenly get better and be able to come home and – and live a full life again…But, I also accept that this is where we are, and that there isn’t anything you can really do to change the situation, other than see him as often as possible, give him the support”.* A daughter of one of the residents gave the following advice in relation to coming to terms with or accepting her situation and connecting to a family member with dementia in a positive way: “*If they’re feeling like things are lost – been lost to them because of having to bring someone here to be looked after because they’re unable to do it themselves any more, uh, to me, you still have to keep positive about it, in that, okay, that’s your mother, your father, your aunt, your uncle, whatever, and – and they’re still there; it’s just that they don’t remember everything like they used to, right? And just because they can’t remember things as they were, you should let them be, because this is the – like, part of their life that they are remembering at that time. That it’s not a bad thing; it’s just something that happens”.*

#### Living life in the moment

One of the daughters expressed how her current situation with her mother in LTC made her more aware of how important it is to live life in the present, as she said: *“it brings greater awareness of how fragile our existence is and how much life needs to be enjoyed in the here and now for we know not when that may be taken from us”.* Living in the moment was also expressed by a spouse who stated that: *“I only live for now, I don’t I don’t plan anything, I don’t – whatever’s happening at the moment”.* The sub-theme “Living in the moment,” suggests that hope as possibilities for a better future may also be defined in moments, as well as months or years.

In contrast to ‘living in the moment’ some participants expressed concern for their own future health in the hope *“that I don’t end up the same way”* as the family member in LTC. This was specifically articulated by one daughter whose parents were both diagnosed with dementia: *“It is certainly my fervent hope that something takes me quickly and painlessly before I become demented”.*

#### Believing in something

For some participants the overarching theme of “hope and connection” also included a connection with their faith. The interconnectedness of hope and faith was expressed by one daughter when she said: *“You must believe for something to have hope…”* For some, the belief in a spiritual being or God helped to maintain hope throughout times of struggle and despair. As one daughter said: “*When I was at my lowest and I remembered that there is a – a spirit or a God out there that loves me unconditionally, and – and that’s what gave me the strength to carry on…and that strength…and hope in my faith that everything has been building and nurturing me. Uh, it- it- it – it keeps me from getting too anxious, even though I will be going through trials and struggles…somehow, it always works out”*. Another daughter derived hope for her father’s well-being related to his faith when she said that: *“I think just knowing my father’s faith gives me hope that he is content”.*

#### Standing together

Several participants spoke of how hope was about connecting with others. For instance, one daughter spoke of the need for the involvement of all family members: “*My hope, is that somehow, there would be some kind of a teaching to show the whole family that they have to stand together in order for it to work well. Because even though there’s a facility for your loved one, it still takes family to keep that loved one content”.* Another daughter spoke of the support she felt from her sisters as they dealt with their father’s deterioration: *“nothing happens but you phone each other and see what’s happening…you don’t feel like you’re all by yourself”.*

Participants also described the importance of developing relationships with staff and other informal caregivers visiting loved ones in the LTC facility. As one spouse said:…*I met R; she’s – her husband was in here, and…she was like a breath of fresh air…so up with everything…the four of us would go downstairs and sit and have coffee or tea…it was the two of us [chuckles], I think, that – that, uh, [we] really got to know each other well…so that’s what I mean about – it’s – like they become your family”.* One of the participants expressed how the staff in LTC and other visitors became part of her family as she said: *“you get to know everybody pretty soon…it’s nice and it sort of becomes like your family”.*

#### Balancing dual worlds

As described previously in the context, the participants’ experience was one of dual worlds where they experienced changes to time and self. Within this context, the participants described how they needed to find balance in their lives so they could continue to have hope and connect with their family member. For example, one participant said that: *“If you don’t have a counterbalance, and, uh, um, I think could very easily become depressed…if not depressed, certainly get a skewed version of, uh, you know, of, uh, of reality”*. Other’s described the importance of balancing dual worlds by engaging in community activities, maintaining relationships with other family members and friends, and continuing to do things they enjoy in life. As one daughter said, *“You have to find it (hope) within yourself, uh, and just what gives you pleasure each day with friends and family”.*

Yet for some, giving themselves permission to enjoy life outside of their commitment to care for a family member in LTC was difficult. For instance, one individual recounted how she used to visit her husband every day and “*couldn’t even think about going*” on a trip with friends for she “*didn’t ever want to feel guilty”.*

## Discussion

The overarching theme of “hope and connection” emphasizes the importance of the relational aspects of hope [13]. A recent meta-synthesis of hope in family caregivers found several studies that described relationships with others as an external factor influencing hope [14]. Connecting with others has also been described as fostering the hope of family caregivers of persons with dementia living at home [3]. However the findings of our study suggest that “hope and connection” is more than an external factor or something that fosters hope. It is an indivisible/dual element. Hope for the participants does not occur without connection and connection is an integral component of hope. There is also reciprocity within “hope and connection”, as the positive action of hope increases positive connection with others and vice versa. This indivisible/ dual element of “hope and connection” is reflected in Farran, Herth and Popovich’s [15] conceptualization of hope. They described the relational processes of hope as the “heart of hope”, suggesting that hope is primarily orientated to some person [15]. In the present study, participants” hope was orientated toward their family member with dementia.

“Hope and connection” exists within a relational space. Jevne [16] writes about this relational space of hope as: “*hope is not something I do for you, it is a place where I meet you.*” (p. 281). Thus hope does not occur unless a person is present in the life of another. The overarching theme of “hope and connection” reflects the residents’ disease processes associated with dementia and the environmental factors associated with having a family or friend residing in a LTC facility. For example dementia interferes with the ability of persons to communicate and connect with others in familiar or traditional ways, thus decreasing the hope of family and friends. Environmental factors associated with having a family or friend residing in a LTC facility, such as physical separation and geographical distance, also interfere with relating with others, impacting hope. The theme of “hope and connection” is unique, as no other published study has reported this finding as a major aspect of the participants’ hope experience.

Several of the sub-themes are similar to other studies of family caregivers of persons’ living with chronic disease. For example, accepting the situation, living in the moment, spirituality and support from others were described in a meta-synthesis study of hope in family caregivers [14]. In the 14 studies included in the meta-synthesis, these themes were described as pathways to hope by study participants. They may also be pathways to hope for family members of persons with dementia in LTC. However, the purpose of the study was not to describe pathways but rather to provide an in-depth description of hope. Further research with different methodologies should be conducted to determine if living in the moment, spirituality and support from others are pathways to hope in this population.

Balancing dual worlds was not reported as a finding in the meta-synthesis of the hope experience of family caregivers of persons with chronic illness [14]. In the hope experience of family caregivers who were bereaved [17] and male spouses of women with breast cancer [18], balancing the positive and negatives of their experiences were described as processes of hope. Both of these groups experienced a daily struggle to find balance, so that negative emotions did not result in despair. This balancing of positive and negative emotions differs from the findings of our study. Family caregivers of persons with dementia residing in LTC described the need to find a balance between their dual worlds. These worlds were both physical and relational. The physical reality of living apart from the person with dementia resulted in dual relational worlds: one encompassed the participants’ relationships with others outside of the facility and the other with their family member in LTC. The dual physical and relational worlds also resulted in changes in self and time for the participants along with waves of hope and hopelessness.

### Factors influencing the study

Several factors influence and limit this study, including the characteristics of the participants and the study design. Most participants were older and well-educated and it is possible that younger family members and those with lower education levels may experience hope differently. The participants represented a diverse group of caregivers in regard to their gender, relationship to the person with dementia, and years of caregiving. They also had relationships with family members in differing stages of dementia. For the purposes of this study, this diversity was intentional as it is an important aspect of interpretive description [12].

As with any qualitative study, the interpretation of the findings must be considered within the context of the lives of the participants. Future research should explore whether the hope experience differs for younger, less educated informal caregivers of persons residing in other facilities.

## Conclusions

Hope was important and essential for family caregivers of persons with dementia residing in a LTC facility. The importance of the relational aspects of hope have previously been alluded to in descriptions of hope, but the main theme of “ Hope and Connection” suggests that for family caregivers of persons with dementia residing in LTC, connecting with the resident is a key element in their hope experience. Future research needs to explore the best ways for “hope and connection” to occur, including the possibility of using innovative approaches such as reminiscence, music, photography and other creative arts. “Hope and connection” and its sub-themes as presented in the findings, have not been described in other studies. With the paucity of hope research focusing on this population, these findings provide a foundation for future research exploring “hope and connection” and the relational space within which this occurs.

### Implications for practice

Although this is an exploratory study, there are several implications for practice. The importance of hope suggests that those working with caregivers need to assess hope and utilize strategies to foster hope in this population. The overarching theme of “hope and connection” emphasizes the importance of maintaining relationships and connection between family members and persons in LTC. Healthcare professionals can suggest strategies to family members/friends to connect with the resident in meaningful ways. Our data suggests that connection can be verbal as well as non-verbal. Policy and decision makers in long term care facilities, also need to consider how policies and the facility environment support continuing relationships and connections between family members and persons residing in the facility. For example, the availability of common meeting areas (eg. atriums, quiet areas, private dining rooms), as well as structured activities (eg. afternoon teas, music programs) can provide opportunities for family members and residents to make meaningful connections and memories.

The sub-themes of accepting the situation, living in the moment, spirituality and support and balancing dual worlds also have implications for health care professionals and those working with family caregivers. These sub-themes could be used as a guide to explore strategies to foster hope in this population. For example health care providers could focus on anticipatory grief issues and strategies to help family members accept their own, as well as the residents’ situation. They could also provide family caregivers with resources and supports that are available to them as caregivers within the facility and in the community. As well they could discuss strategies to balance family caregivers’ dual worlds, emphasizing the important of self-care and helping them set limits around the amount of care family caregivers’ provide. The findings of this study could also be shared with other caregivers, in support groups for family caregivers and in training programs for health care professionals working with people with dementia. Sharing the participants’ insights from this study with other family caregivers who might be undergoing similar experiences, may help normalize their situation and potentially enhance their hope experience and well- being. Future research needs to be directed towards the evaluation of the effectiveness of these strategies in fostering hope for family caregivers of persons with dementia residing in LTC.

## Competing interests

The authors declared that they have no competing interests.

## Authors’ contributions

WD and CN conceptualized the study. WD was responsible for the overall study. DS collected the data and completed a draft report of the findings. All authors contributed to the analysis and interpretation of the data. WD drafted the manuscript. DS and CN critically commented on and approved the final manuscript. All authors read and approved the final manuscript.

## Pre-publication history

The pre-publication history for this paper can be accessed here:

http://www.biomedcentral.com/1471-2318/13/112/prepub
